# Codeine and promethazine: Exploratory study on “lean” or “sizzurp” using national survey data and an online forum

**DOI:** 10.1371/journal.pone.0301024

**Published:** 2024-03-25

**Authors:** Orrin D. Ware, Albert Garcia-Romeu, C. Austin Zamarripa, Tamera Hughes, Leeza Wager, Tory Spindle

**Affiliations:** 1 School of Social Work, University of North Carolina at Chapel Hill, Chapel Hill, NC, United States of America; 2 School of Medicine, Johns Hopkins University, Baltimore, MD, United States of America; 3 School of Pharmacy, University of North Carolina at Chapel Hill, Chapel Hill, NC, United States of America; NYU Grossman School of Medicine: New York University School of Medicine, UNITED STATES

## Abstract

**Background:**

The concoction known as "lean" containing codeine and promethazine, holds a prominent cultural presence and is often referenced in mass media platforms (e.g., music and social media). Surprisingly, there’s a scarcity of national data characterizing the use of lean. Therefore, the current study investigated the use of lean using national survey data and online forum participant input, and focused on identifying concurrent substance use, exploring co-administration with other substances (e.g., alcohol, cannabis), and determining lean-related experiences.

**Methods:**

We analyzed data from the National Survey on Drug Use and Health (NSDUH) spanning 2007–2019, identifying persons who used lean (weighted N = 42,275). Additionally, we conducted a Reddit-based study to gather insights about lean consumtion (N = 192).

**Results:**

The NSDUH data indicated that lean use was most prevalent among teenagers and young adults (ages 13–21), accounting for 66% of the sample. This trend was more pronounced in male respondents (75%) compared to females. Additionally, the use was predominantly observed among Black/African American (29%), Hispanic (28%), and White (33%) populations, with these groups also reporting higher levels of concurrent alcohol and cannabis use. Similarly, findings from Reddit showed that individuals who used lean were predominantly male (67%) and exhibited elevated concurrent rates of alcohol (83%) and cannabis (46%) use in the past 30 days. Moreover, approximately 66% of respondents met criteria for severe lean use disorder, and 37% acknowledged driving under its influence.

**Conclusion:**

The NSDUH data found that mostly young adult males reported consuming lean in the past twelve months, though the racial/ethnic breakdown of persons who used lean was diverse. The Reddit data found that most individuals in the sample met the criteria for a substance use disorder pertaining to their lean consumption. These findings underscore the clinical significance and necessity for further controlled research on lean.

## Introduction

The combination of codeine and promethazine often describes ingredients contained in the liquid concoction colloquially called “lean” or “purple drank” or “sizzurp” (referred to as lean hereafter) [1–5]. Despite its significant cultural presence [4, 6–10], there is a lack of studies examining national data concerning the use of lean [1, 11]. This concoction has achieved remarkable cultural prominence, as evidenced by its frequent mention in Billboard Hot 100 songs, even those peaking within the top 10 position. Some notable examples include Lil Nas X’s 2019 “Old Town Road”, which peaked at the first position for nineteen weeks (i.e., “Lean all in my bladder”) [12], Roddy Ricch’s 2019 “The Box”, which peaked at the first position for eleven weeks (i.e., “Pour up the whole damn seal, I’ma get lazy”) [13], Internet Money’s 2020 “Lemonade”, which peaked at the sixth position (i.e., “Off the juice, codeine got me trippin’”) [14], and Gunna’s 2023 “fukumean”, which peaked at the fourth position and currently ranked seventh at the time of drafting this article, September 2023 (i.e., “I’m about to pour up some syrup”) [15]. Given the undeniable cultural significance of lean, studies using national data and primary data collection to describe lean use are warranted [1, 11]. It is also important to examine the different pharmaceutical indications of both codeine and promethazine.

Codeine, historically obtained naturally from opium poppy plants but now synthesized from synthetic morphine, has long been used as a pain reliever and cough suppressant [1, 16]. The availability of codeine-containing products varies by region and country. In some places, certain codeine-containing products are available over the counter without a prescription, while others require a prescription due to their potential for misuse and addiction [17]. Promethazine is a first-generation antihistamine that helps in alleviating allergy symptoms, such as sneezing, itching, and runny nose. Additionally, promethazine possesses anticholinergic properties, contributing to its sedative and antiemetic effects [18]. Promethazine is frequently used in cough syrups due to its sedative and central nervous system depressing properties. When combined with codeine, it serves as one of the primary treatments for providing temporary relief from coughs and upper respiratory symptoms linked to allergies or the common cold in individuals aged 18 and older [19]. Long-term use of promethazine and codeine is generally discouraged due to the drugs’ adverse effects of tolerance, dependence, and addiction. These potential risks have not gone unnoticed with agencies responsible for public health such as the United States Food and Drug Administration (FDA) issuing guidance. In April 2023 as part of their *Updates to Opioid Prescribing Information*, the FDA issued a drug safety communication to announce safety-related updates to the prescribing information for all opioid analgesics, including codeine [20].

Surprisingly, there is limited research on lean. A systematic review of this substance combination found only seven studies meeting the inclusion criteria, which were (a.) papers focused on the clinical and social implications of lean, (b.) article types that were either case reports, case series, open label or double-blind trials, original articles, prospective or retrospective observational studies, (c.) articles written in English or French, and (d.) excluded papers which were: animal/in vitro experiments, commentaries, meta-analyses, reviews, or substances not directly called “Purple drank” [1]. While the authors of the systematic review used the search string “*Purple AND drank*” [1], similarly, using the search string “*Codeine AND Promethazine*” for the years 1963 to 2022 yields few results in PubMed with only n = 71 results (of which many articles do not describe the combined use of these substances recreationally). Furthermore, data regarding the prevalence of lean use are sparse, with one study reporting that approximately 7% of students in a southeastern U.S. college self-reporting consuming lean [21], and another study finding that approximately 16% of surveyed electronic dance music party attendees in New York City had at least one life-time use of lean [5]. While these findings offer insights into lean use among specific demographic groups and geographic regions, there remains a significant gap in the literature concerning national data on patterns of consumption [1, 11]. These gaps in the literature highlight the limited clinical understanding of lean, including its potential health impacts, and possible avenues for therapeutic intervention.

Another notable gap in the literature concerns the investigation of substances commonly co-consumed with lean, a phenomenon often referred to as co-use or polysubstance use (when multiple substances are used together) [22–27]. Alcohol and cannabis are two of the most used substances (excluding caffeine and nicotine/tobacco) [28], with approximately 47.5% of individuals in the U.S. reporting alcohol use, and 18.8% reporting cannabis use in 2021 [28]. Notably, one study exploring lean consumption among electronic dance music party attendees found that of the 41 individuals who consumed lean in the past year, 33 (80%) also consumed cannabis within the same year. Prior research has documented instances of alcohol and lean co-use, including cases where alcohol is mixed directly into lean [1]. Additionally, a content analysis of 40 songs referencing lean found that many of these songs described co-use with alcohol or cannabis [6]. It is also worth noting that no extant studies have characterized lean-related substance use disorder (SUD); SUD is defined as the problematic pattern of using a substance that meets at least two diagnostic criteria within a twelve-month period, such as craving, tolerance, or withdrawal (withdrawal is physiological or psychological symptoms experienced after the decreased use of a substance that was previously used heavily or regularly) [29].

The present exploratory study aims to gaps in the literature about lean by utilizing data from a national survey (i.e., National Survey on Drug Use and Health (NSDUH)) and from participants recruited from an online forum (i.e., Reddit). The primary objectives of this study are threefold: (1) to examine co-use of alcohol with lean, (2) to identify co-use of cannabis with lean, and (3) to investigate experiences related to lean use (i.e. driving under the influence, symptoms indicative of SUD including withdrawal). By addressing these gaps in the literature, this study will provide needed data on lean use patterns and lean use experiences, which in turn can be leveraged to help minimize public health risks, and enhance our knowledge-base for treating individuals presenting with problematic lean use in medical settings.

## Materials and methods

This study used data from two datasets, one publicly available national dataset from NSDUH public use file and another with primary data collected from individuals on social media using Reddit. Using data from the NSDUH allowed us to examine demographic characteristics and substance use patterns among a large national sample of individuals who self-reported lean use. Data from the Reddit Lean Use Study provided the opportunity to capture more specific data about lean use experiences such as lean co-used with alcohol and lean co-used with cannabis which are not available in the NSDUH. Descriptions of both datasets may be found below. The University of North Carolina at Chapel Hill Institutional Review Board determined the study procedures using the publicly available de-identified NSDUH to be non-human subjects research (Principal Investigator: Ware; Study Number: 22–1833). Procedures for the human subjects research involved in the data collection and analysis of the Reddit Lean Use Study dataset were approved by the University of North Carolina at Chapel Hill Institutional Review Board (Principal Investigator: Ware; Study Number: 22–2509).

### National survey on drug use and health

The NSDUH is an annual survey led by the Substance Abuse and Mental Health Services Administration that captures national and state-level data about health topics, including past year substance use, past month substance use, and past year substance use disorders in the US [30]. Using scientific methods, households are randomly selected to participate in the survey. Data are collected from persons ages 12 years and older. After applying sample weights, the survey estimates are representative of the US population.

The current study used the de-identified publicly available NSDUH 2002–2019 public use file [30]. The NSDUH 2002–2019 public use file merges the cross-sectional survey data across these years (2002 to 2019). This dataset is accompanied by a codebook that provides the meaning of each variable and responses for each variable. A content analysis of the codebook was conducted to examine the mention of “codeine” and “promethazine.” These descriptions were further reviewed to identify “codeine” and “promethazine” being co-used in the past 12 months to identify persons who ingested lean. These instances were identified as responses in the following four variables “COLDYR1”, “COLDYR2”, “COLDYR3”, and “COLDYR4” [30]. Each of these four variables allowed persons to specify which cough/cold medicine they used in the past 12 months as a follow-up. The content analysis of the codebook (searching for the words “lean”, “codeine”, and “promethazine” separately) identified the following response items about lean in the past 12 months, “Lean (Promethazine w/codeine mixed w/Sprite),” “Phenergan w/Codeine, Promethazine w/Codeine,” and “Promethazine VC with Codeine.”

Respondents were included in the current study as using lean in the past 12 months based on the following inclusion criteria: (a) selected Yes to ever “TAKEN A NONPRESCRIPTION COUGH MED TO GET HIGH,” (b) selected within the past 12 months to “TIME SINCE LAST USED COUGH/COLD MED,” and (c) selected “Lean (Promethazine w/codeine mixed w/Sprite),” “Phenergan w/Codeine, Promethazine w/Codeine,” or “Promethazine VC with Codeine” to “OTHER COUGH/COLD MED USED PAST 12 MOS—SPECIFY” [30]. The sample included 219 individuals with years of data collection ranging consecutively from 2007–2019. Using the thirteen-year sample weight variable in the dataset, estimates for 42,275 persons were produced.

Variables used in the current descriptive study included: (a) year of data collection (2007–2019), (b) age (categorical) (c) gender, (d) race and ethnicity, (e) past 12 month substance use (Yes/No; alcohol, cannabis, cocaine, crack, heroin, and phencyclidine [PCP]), (f) past 30 day substance use (Yes/No; alcohol, cannabis), (g) alcohol use disorder in the past 12 months, and (h) cannabis use disorder in the past 12 months. A description of this sample may be found in Table 1.

**Table 1:** Characteristics of the national survey on drug use and health one year weighted sample with 12 month codeine and promethazine use (lean/sizzurp).

Characteristics	n	%
**Sample Size**	42,275	100.0
**Year of Data Collection**		
2007	799	1.9
2008	1,176	2.8
2009	1,844	4.4
2010	3,231	7.6
2011	2,148	5.1
2012	5,071	12
2013	3,533	8.4
2014	3,842	9.1
2015	2,925	6.9
2016	6,562	15.5
2017	3,612	8.5
2018	4,038	9.6
2019	3,494	8.3
**Age**		
13 to 17 years old	12,388	29.3
18 to 21 years old	15,458	36.6
22 to 25 years old	6,801	16.1
26 to 29 years old	2,209	5.2
30 to 34 years old	2,184	5.2
35 to 49 years old	2,596	6.1
50 to 64 years old	0	0.0
65 years or older	639	1.5
**Gender**		
Female	10,585	25.0
Male	31,690	75.0
**Race and Ethnicity**		
Asian	3,230	7.6
Black or African American	12,169	28.8
Hispanic or Latino of any Race	11,803	27.9
Native Hawaiian/Other Pacific Islander	85	0.2
Native American/Alaska Native	249	0.6
Two or More Races	887	2.1
White	13,852	32.8
**Past 12 Month Use** ^**1**^		
Alcohol	39,183	92.7
Cannabis	36,338	86.0
Cocaine	11,971	28.3
Codeine and Promethazine (Lean/Sizzurp)	42,275	100.0
Crack	108	0.3
Heroin	1,103	2.6
PCP	502	1.2
**Alcohol and Cannabis** ^**1**^		
Alcohol use (Past 30 days)	33,524	79.3
Cannabis use (Past 30 days)	32,093	75.9
Alcohol use Disorder (Past 12 months)	13,341	31.6
Cannabis use Disorder (Past 12 months)	15,579	36.9
Alcohol and Cannabis Use Disorder (Past 12 months)	7,244	17.1

Thirteen Year Weighted data from a sample of n = 219.

Some percentages may not equal 100.0% due to rounding.

^1^These categories under this heading are not mutually exclusive.

### Reddit lean use study

The Reddit Lean Use Study recruitment procedures have been described elsewhere [11]. The popular social media online discussion forum Reddit was used to recruit adults who have ever used lean to complete a one-time 20-minute anonymous online survey. Through Reddit, the study PI (Ware), messaged moderators of subReddits (self-created communities, united by a certain topic) that focused on music or substances. The moderators were asked for permission to post study recruitment information including the study’s purpose, eligibility, survey link, survey QR code, PI contact information, and IRB approval information. After receiving approvals from moderators, the PI posted recruitment materials approximately once per week from October 2022 to November 2022 to seven subReddits that focus on substances.

The survey was completed via Qualtrics [31] with a recruitment period of October 25, 2022, to January 4, 2023. Qualtrics bot detection features were activated for the survey [31]. After accessing the survey, respondents were directed to the first page, which in text described the study, described the voluntary nature of the research, described rights of research participants, and asked if they were interested. Respondents who selected ≥18 as their current age, selected “Yes” to “Have you ever used lean? Sometimes it is called purple, purple drank, double cup, sizzurp, or dirty sprite,” and responded correctly to a quality check question were eligible to complete the survey. Respondents were notified of their eligibility status, and ineligible persons were thanked for their time. Eligible respondents were asked if they were interested in completing the survey. Participants who completed the survey were given a four-digit code that would be entered into a separate survey alongside their e-mail address to enter a drawing based on chance in which 20 individuals who completed the survey would receive a $50 virtual gift card.

There were 2,883 responses in the initial dataset downloaded from Qualtrics. However, after applying the bot detection feature, selecting eligible respondents, selecting persons who reported lean use in the past 12 months, selecting persons who responded to eleven items about lean use disorder, selecting individuals who include codeine as an ingredient in their lean, and selecting individuals who include promethazine as an ingredient in their lean, this sample included N = 192 persons. A previous study using this dataset to examine the association between lean use and coping with mental health symptoms included 1,423 persons who selected using either codeine or promethazine as an ingredient [11]. The decision was made to include different variations of reported codeine or promethazine in the previous study [11] to better understand self-reported lean ingredients since there may be variability in the ingredients in lean [5]. However, the current study examined the subsample of N = 192 persons who reported including codeine and promethazine as ingredients in their lean to mirror the ingredients described in the NSDUH (as described in the National Survey on Drug Use and Health section above). A description of this sample may be found in Table 2.

**Table 2:** Characteristics of the reddit sample with past 12 month codeine and promethazine use (lean/sizzurp).

Characteristics	n	%
**Sample Size**	192	100.0
**Age, Mean (SD)**	27.4 (5.2)	
**Gender**		
Female	58	30.2
Male	129	67.2
Another Gender Identity	5	2.6
**Race and Ethnicity**		
Black or African American	10	5.2
Hispanic or Latino of any Race	79	41.1
White	83	43.2
Another Race or Ethnicity	20	10.4
**Past 12 Month Use** ^**1**^		
Alcohol	183	95.3
Cannabis (Past six months)	98	51.0
Cocaine/Crack	59	30.7
Codeine and Promethazine (Lean/Sizzurp)	192	100.0
Heroin	18	9.4
PCP	12	6.3
**Alcohol and Cannabis** ^**1**^		
Alcohol use (Past 30 days)	159	82.8
Cannabis use (Past 30 days)	89	46.4
**Lean Use Disorder (LUD)**		
No LUD	16	8.3
Mild LUD	15	7.8
Moderate LUD	34	17.7
Severe LUD	127	66.1
**Lean/Sizzurp** ^**1**^		
Lean/sizzurp use (Past 30 days)	170	88.5
Lean/sizzurp co-use with alcohol (Past 30 days)	113	58.9
Lean/sizzurp co-use with cannabis (Past 30 days)	54	28.1
Experienced lean/sizzurp withdrawal (Ever)	142	74.0
Overdosed from lean/sizzurp (Ever)	120	62.5
Had seizures from lean/sizzurp (Ever)	68	35.4
Received treatment for lean/sizzurp use (Ever)	105	54.7
Wanted treatment for lean/sizzurp (Ever; n = 85 respondents)^2^	36	18.8
Ever driven a vehicle during or after drinking lean/sizzurp	70	36.5

Some percentages may not equal 100.0% due to rounding.

^1^These categories under this heading are not mutually exclusive.

^2^42.4% of n = 85 respondents.

Variables in the current study included (a) age (continuous) (b) gender (female, male, other gender identity), (c) race and ethnicity, (d) past 12 month substance use (Yes/No; alcohol, cannabis, cocaine/crack, heroin, and PCP), (e) past 30 day substance use (Yes/No; alcohol, cannabis), (f) alcohol use disorder in the past 12 months, and (g) cannabis use disorder in the past 12 months.

Other variables assessing experiences with lean were added. These variables were:

- past 30 day lean use (“Have you used lean in the last 30 days?” Yes/No),
- past 30 day lean co-use with alcohol (“In the last 30 days have you used lean and alcohol together?” Yes/No)
  - Note: This was asked if participants (a) selected Yes to using lean in the past 30 days, (b) selected Yes to using alcohol in the past 30 days, and (c) selected Yes to “Have you **ever** used lean and alcohol together?”),
- past 30 day lean co-use with cannabis (“In the last 30 days have you used lean and cannabis together?” Yes/No)
  - Note: This was asked if participants (a) selected Yes to using lean in the past 30 days, (b) selected Yes to using cannabis in the last 30 days, and (c) selected Yes to “Have you **ever** used lean and cannabis together?),
- ever experienced lean withdrawal (“Have you ever experienced withdrawal from lean?” Yes/No)
- ever overdosed from lean (“Have you ever overdosed from lean?” Yes/No),
- ever had seizures from lean (“Have you ever had seizures from lean?” Yes/No),
- ever received treatment for lean use (“Have you ever received treatment for lean use?” Yes/No)
- ever wanted treatment for lean (“Have you ever wanted treatment for lean use?” Yes/No)
  - Note: This was asked if participants selected No to ever receiving treatment for lean use
- ever driven a vehicle during or after drinking lean (“Have you ever driven a vehicle during or after recently drinking lean?” Yes/No)

A lean use disorder measure [11] was captured by using eleven items that were adapted from the Diagnostic and Statistical Manual of Mental Disorders Fifth Edition Text Revision’s (DSM-5-TR) eleven substance use disorder criteria [29]. Respondents could select Yes = 1 or No = 0 to the eleven items which are described elsewhere [11]. After summing these items, four categorical groups were identified, (a) no lean use disorder (score: 0–1), (b) mild lean use disorder (score: 2–3), (c) moderate lean use disorder (score: 4–5), and (d) severe lean use disorder (score: ≥ 6). The Cronbach’s alpha for this sample was .797.

The Alcohol Use Disorders Identification Test-Concise (AUDIT-C) was used to examine at-risk alcohol consumption [32]. The AUDIT-C is a three-item measure with scores ranging from 0 to 12, with a score of ≥ 3 being indicative of at-risk alcohol consumption for women and a score of ≥ 4 being indicative of at-risk alcohol consumption for men. For the purpose of this study, the cutoff of ≥ 3 was used for persons who self-reported as Another gender identity. The Cronbach’s alpha for this sample was .533.

The Cannabis Use Disorder Identification Test-Revised (CUDIT-R) was used to examine at-risk cannabis use [33]. The CUDIT-R is an eight-item measure with scores ranging from 0 to 32. After responding to a lead-in question that assesses cannabis use in the past six months (Yes/No), individuals were asked the eight items. After summing these items three categorical groups were identified, (1) non-hazardous cannabis use (score: 0–7), (2) hazardous cannabis use (score: 8–11), and (3) possible cannabis use disorder (score: ≥ 12) [34]. The Cronbach’s alpha for this sample was .743.

### Data analysis

All data analyses were conducted using IBM SPSS Statistics Version 28.0 [35]. Figures were created using R [36], with the package “ggplot2” [37] and “scatterplot3d” [38]. Using the “WEIGHT” command in SPSS, a thirteen year sample weight variable that is found in the dataset was applied to the NSDUH sample since the data included in the analyses covered thirteen years, 2007–2019. Univariate statistics were conducted to describe the NSDUH sample.

Univariate statistics were conducted to describe the Reddit Lean Use Study sample. Using data from the Reddit Lean Use Study an exploratory linear regression model examined the effect of demographic characteristics (1) age (continuous), (2) gender (Female/Woman, Another Gender Identity, and Reference Group: Male/Man), and (3) race/ethnicity (Black or African American, Hispanic or Latino any race, Another Race or Ethnicity, and Reference Group: White) on the lean use disorder measure (continuous). The data were examined and met all the assumptions for the multivariable linear regression model. Results were considered significant at p < .05.

## Results

### National survey on drug use and health lean sample results

As seen in Table 1, the NSDUH sample of individuals reporting past-year use of lean were primarily male (75.0%). Alcohol was used by 92.7% of the sample in the past 12 months and 79.3% of the sample in the past 30 days. Alcohol use disorder was identified in 31.6% of the sample. Cannabis was used by 86.0% of the sample in the past 12 months and 75.9% of the sample in the past 30 days. Cannabis use disorder was identified in 36.9% of the sample. The co-occurrence of alcohol and cannabis use disorder was identified among 17.1% of individuals in the sample of individuals who used lean in the past year.

### Reddit lean use study sample results

Of the N = 192 individuals recruited from Reddit, 54 (28.1%) added “Other Ingredients” with their codeine and promethazine lean mixture. After respondents selected “Other Ingredients” they were asked to specify these ingredients in an open-ended text box. Ingredients that were identified more than once in these open-ended responses included (1) soda/soft drink (*n* = 18), (2) other opioid (*n* = 7), (3) alcohol (*n* = 4), (4) benzodiazepine (*n* = 4), (5) cannabis (*n* = 4), (6) candy (*n* = 3), and other antihistamine (*n* = 2).

As seen in Table 2, the Lean Use Reddit Study sample were primarily male (67.2%). Alcohol was used by 95.3% of the sample in the past 12 months and 82.8% of the sample in the past 30 days. Cannabis was used by 51.0% of the sample in the past six months and 46.4% of the sample in the past 30 days. Regarding substance co-use, 58.9% of the sample co-used alcohol and lean in the past 30 days, and 28.1% of the sample co-used cannabis with lean in the past 30 days. Further, 23.4% persons self-reported both co-use of alcohol with lean and co-use of cannabis with lean in the past 30 days.

**Fig 1:**
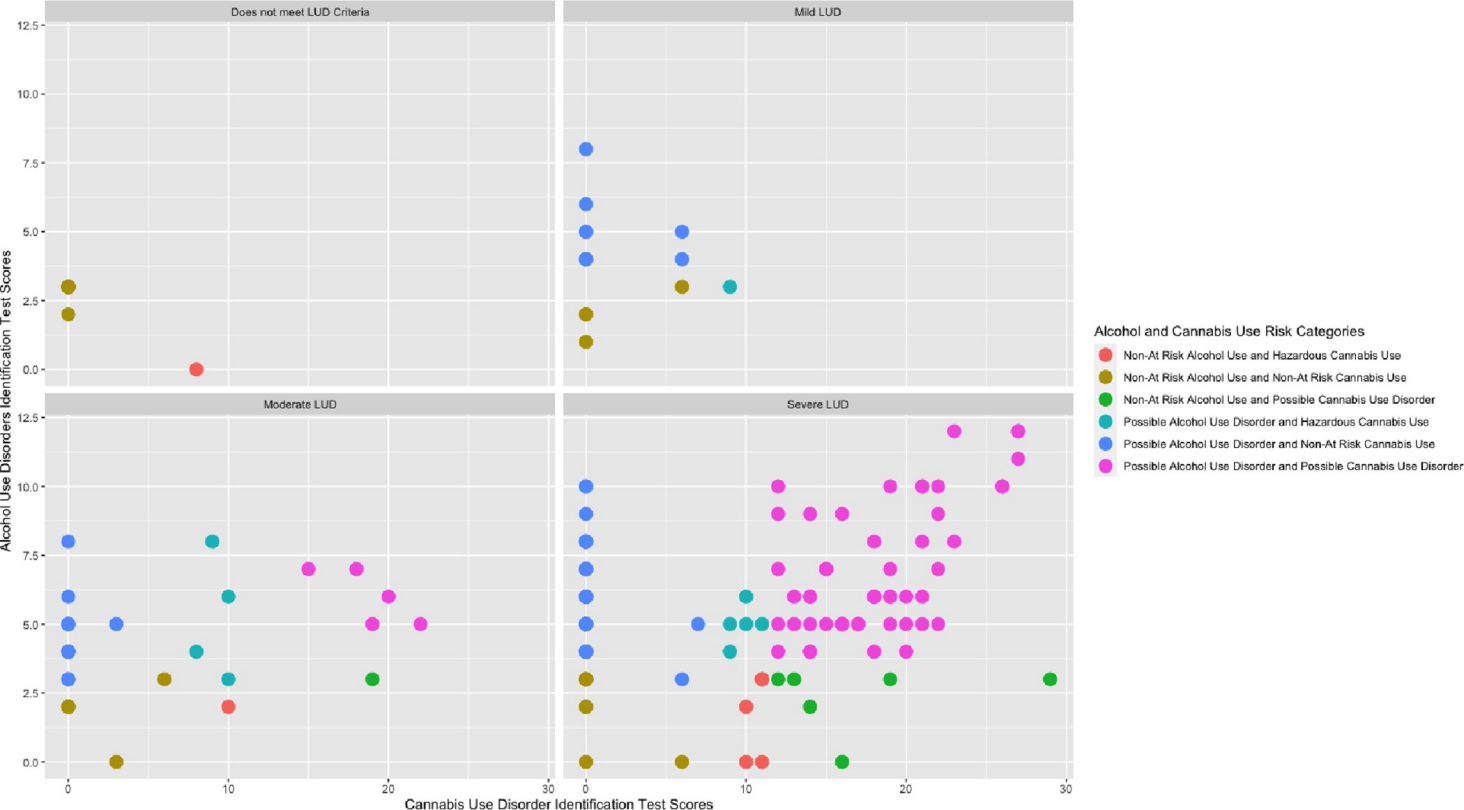
Alcohol and cannabis risk scores among lean use disorder (LUD) groups. Note: N = 190 (n = 2 were missing data). The Alcohol Use Disorders Identification Test Concise has possible scores ranging from 0 to 12, with a score of ≥ 3 being indicative of at-risk alcohol consumption for women (and for persons who self-reported as Another gender identity) and a score of ≥ 4 being indicative of at-risk alcohol consumption for men. The Cannabis Use Disorder Identification Test Revised has as range of possible scores ranging from 0 to 32 with 0 to 7 indicating non-hazardous cannabis use, 8 to 11 indicating hazardous cannabis use and ≥ 12 indicating possible cannabis use disorder. The Lean Use Disorder Measure has possible scores ranging from 0 to 11 with 0 to 1 indicating no lean use disorder, 2 to 3 indicating mild lean use disorder, 4 to 5 indicating moderate lean use disorder, and ≥ 6 indicating severe lean use disorder.

Most persons in the sample met the criteria for severe lean use disorder at 66.1%. Fig 1 shows at-risk use for alcohol and cannabis among the four different lean use disorder groups. A total of 138 (71.9%) persons met the AUDIT-C cutoff for at-risk alcohol consumption. Considering at-risk cannabis use, 9.4% of persons met the cutoff for hazardous cannabis use and 34.4% met the cutoff for possible cannabis use disorder using the CUDIT-R.

**Fig 2:**
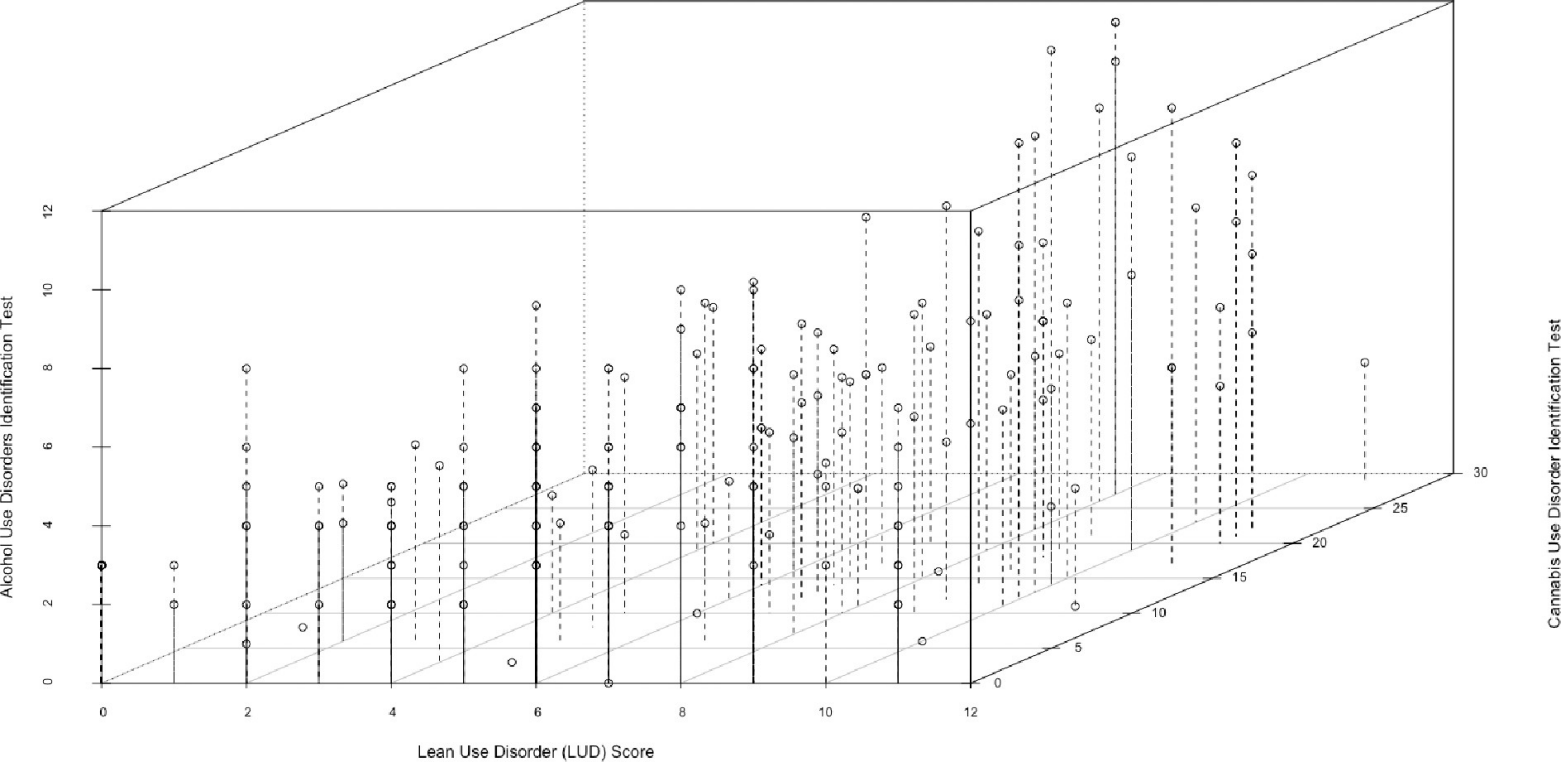
3D Scatterplot of lean, alcohol, and cannabis use disorder screening scores. Note: N = 190 (n = 2 were missing data). The Alcohol Use Disorders Identification Test Concise has possible scores ranging from 0 to 12, with a score of ≥ 3 being indicative of at-risk alcohol consumption for women (and for persons who self-reported as Another gender identity) and a score of ≥ 4 being indicative of at-risk alcohol consumption for men. The Cannabis Use Disorder Identification Test Revised has as range of possible scores ranging from 0 to 32 with 0 to 7 indicating non-hazardous cannabis use, 8 to 11 indicating hazardous cannabis use and ≥ 12 indicating possible cannabis use disorder. The Lean Use Disorder Measure has possible scores ranging from 0 to 11 with 0 to 1 indicating no lean use disorder, 2 to 3 indicating mild lean use disorder, 4 to 5 indicating moderate lean use disorder, and ≥ 6 indicating severe lean use disorder.

Fig 2 shows a scatterplot of the total scores for the AUDIT-C [32], CUDIT-R [33], and lean use disorder measures [11]. The means and standard deviations (SD) for these measures include 5.2 (SD = 2.3) for the AUDIT-C (n = 183), 15.3 (SD = 5.8) for the CUDIT-R (n = 93), and 4.4 (SD = 2.4) for the lean use disorder measure (N = 192).

Table 3 shows the results of the multivariable linear regression model assessing the relationship between participant demographic characteristics and lean use disorder. The model was not significant (F(6, 185) = 1.685, p = .127), with an R^2^ of .052.

**Table 3:** Effect of demographic characteristics on lean use disorder symptoms.

Independent Variable	b	95% CI	beta	t	p
(Constant)	5.20	2.77, 7.64		4.22	< .001
Age	0.02	-0.07, 0.10	0.03	0.46	.647
Gender (Reference: Male/Man)					
Female/Woman	0.24	-0.73, 1.21	0.03	0.49	0.628
Another Gender Identity	-1.55	-4.35, 1.26	-0.80	-1.09	.278
Race/Ethnicity (Reference: White)					
Black or African American	1.44	-0.59, 3.47	0.10	1.40	.162
Hispanic or Latino any race	1.35	0.38, 2.33	0.22	2.73	.007
Another Race or Ethnicity	0.94	-0.57, 2.45	0.09	1.23	.221

R-squared: .052 & Adjusted R-squared = .021

## Discussion

Consumption of codeine and promethazine (a concoction colloquially referred to as lean) is prominent in popular culture, but there has been little formal research on this mixture of substances. The present study utilized a national survey (NSDUH) and online discussion forum (Reddit) to attempt to fill very basic gaps in knowledge regarding lean consumption. Specifically, this study quantified basic demographics among those who recently used lean, examined self-reported lean use in the past 12 months (both alone and in combination with cannabis and alcohol), and explored the potential for lean use disorder. Overall, as discussed below, our findings have important clinical implications and highlight the need for additional controlled research on lean.

This study confirmed the findings of other studies that lean consumption is not indicative of any specific demographic group [1, 21]. Further, our linear regression model which examined whether participant demographic characteristics predicted lean use disorder severity screening was non-significant. These findings are important and have direct clinical relevance because popular beliefs about lean consumption primarily focus on young African American males (which could be due to media and specific styles of music) [21]. In this case, null findings indicate that despite depictions in media, lean use may be a phenomenon that cuts across multiple ethnic, gender, and racial groups. Instead of focusing on specific demographic groups, more research should be conducted to identify potential clinical profiles of persons who may be at risk for developing a substance use disorder due to lean consumption.

The present study found high proportions of past year use of cannabis and alcohol among persons who used lean in the past-year. Further, approximately 60% of the sample co-used lean and alcohol, and 28% co-used cannabis with lean in the past 30 days. Prior research on the co-use of other drugs (e.g., cannabis and alcohol; cannabis and tobacco) highlights that individuals engage in co-use for a variety of reasons including to boost/enhance intoxication (i.e., synergistic effects), social influences (e.g., different social settings), and experimentation, among other reasons. Additional research is needed to delineate why individuals may elect to co-use lean with alcohol, cannabis, or other substances and whether co-use (versus solo use of lean) is associated with greater negative clinical and behavioral outcomes. Furthermore, the pharmacological interactions between opioid (i.e., codeine) and antihistamine (e.g., promethazine) medications necessitate further study to delineate the combined effects of these substances, as well as potential interactions with other commonly used substances including alcohol, cannabis, and medications such as benzodiazepines. There is a high likelihood that these combinations may lead to an increased risk of experiencing adverse outcomes such as overdose that could have a substantial public health impact.

This study underscores critical clinical considerations regarding lean consumption. The data revealed that over one-third of the Reddit sample had engaged in driving while under the influence of lean, highlighting a concerning trend. Furthermore, there have been descriptions of consuming lean in conjunction with driving in music [6]. Many respondents in the Reddit Lean Use Study reported ever experiencing withdrawal symptoms (74%), overdoses (63%), or seizures (35%) linked to their lean consumption, mirroring findings from prior studies highlighting potential adverse events associated with high-risk lean use [1, 3, 39–42]. Additionally, a notable number of individuals in the Reddit sample met the criteria for a lean use disorder (91%), potentially indicating the presence of two separate substance use disorders due to the opioid and phenothiazine derivative components.

Considering how absent lean is in the academic literature, readily available clinical guidelines are needed to direct clinicians or providers on treating or providing harm reduction services for persons who may be experiencing the harmful effects of lean. However, the fact that over 50% of the Reddit sample ever received treatment for lean use is promising as it is higher than the typical rates of treatment receipt for other substance use disorders [28]. However, the Reddit sample is not indicative of the general population as this was a select group of persons who responded to the study recruitment material posted to an online substance-related forum. Findings from this study should be considered alongside preliminary evidence that suggests that persons with high-risk lean consumption should also be screened for negative mental health symptoms to ensure that if a co-occurring disorder is identified, treatment may be provided [11, 43].

Although this study provided important insights into the consumption of lean and associated consequences of use, there were several noteworthy limitations. Firstly, while the NSDUH is a national survey, individuals are asked directly about taking a cough or cold medicine to get high and not directly about lean. Instead, lean was specified as a follow-up question about the specific cough or cold medicine used to get high. Therefore, this study is not able to provide the prevalence of lean consumption, which is likely higher since persons were not directly queried from the outset about lean. As this study used multiple waves of annual data from the NSDUH, considerations impacting the potential reliability of the results must be considered alongside the survey’s methodology, which is described elsewhere [30]. Another limitation is use of lean that would meet the criteria for a SUD were not examined in the NSDUH. While limitations from the Reddit study have been described elsewhere [11], some limitations include people recruited from Reddit not being generalizable to a larger population (therefore, findings should not be generalized beyond the respondents), the risk of persons responding in a biased manner, and no geographic data were captured from respondents. Further, the lean use disorder screening variable is still exploratory, although it was developed from the substance use disorder criterion of the Diagnostic and Statistical Manual of Mental Disorders Fifth Edition Text Revision [29]. Another limitation is both datasets are self-reported, which presents the potential for biased responses impacting the reliability of findings. Further, the self-report nature of the Reddit Lean Use Study allows for differences in interpretation when respondents are answering questions such as whether they ever overdosed or ever experienced withdrawal from lean.

Despite these limitations, this study enhances the literature regarding what is currently known about lean. As highlighted elsewhere, findings from this exploratory study should be used to provide a foundational knowledgebase for clinical/research purposes and not to stigmatize individuals who consume lean [11]. Despite the limitations identified, the parallel use of a national survey and online forum in this paper provide a great addition to the research literature regarding lean.

## Conclusion

This study addressed gaps in the literature by identifying lean use patterns and lean use experiences by using national survey data and data collected from an online forum. Findings from the study highlight the potential for future research avenues, including identifying specific subjective experiences of physiological and psychological withdrawal symptoms associated with lean use, subjective responses related to using lean with other substances, clinical profiles of persons who may be at risk of developing a substance use disorders related to their lean use, and potential harm reduction/treatment strategies for individuals with a potential problematic pattern of lean consumption. Further, we must ensure that any clinical or research efforts focusing on individuals who consume lean are done in a non-stigmatizing manner.
